# Dysregulation of SAA1, TUBA8 and Monocytes Are Key Factors in Ankylosing Spondylitis With Femoral Head Necrosis

**DOI:** 10.3389/fimmu.2021.814278

**Published:** 2022-01-18

**Authors:** Jie Jiang, Xinli Zhan, Tuo Liang, Liyi Chen, Shengsheng Huang, Xuhua Sun, Wenyong Jiang, Jiarui Chen, Tianyou Chen, Hao Li, Yuanlin Yao, Shaofeng Wu, Jichong Zhu, Chong Liu

**Affiliations:** The First Clinical Affiliated Hospital of Guangxi Medical University, Nanning, China

**Keywords:** ankylosing spondylitis, immune cell infiltration, immune cell composition analysis, femoral head necrosis, protein park analysis, weighted gene co-expression network analysis, drug sensitivity analysis, routine blood tests

## Abstract

**Introduction:**

The mechanism of ankylosing spondylitis with femoral head necrosis is unknown, and our study aimed investigate the effects of genetic and immune cell dysregulation on ankylosing spondylitis.

**Materials and Methods:**

The protein expression of all ligaments in ankylosing spondylitis with femoral head necrosis was obtained using label-free quantification protein park analysis of six pairs of specimens. The possible pathogenesis was explored using differential protein analysis, weighted gene co-expression network analysis, recording intersections with hypoxia-related genes, immune cell correlation analysis, and drug sensitivity analysis. Finally, routine blood test data from 502 AS and 162 healthy controls were collected to examine immune cell differential analysis.

**Results:**

SAA1 and TUBA8 were significantly expressed differentially in these two groups and correlated quite strongly with macrophage M0 and resting mast cells (P < 0.05). Routine blood data showed that monocytes were significantly more expressed in AS than in healthy controls (P < 0.05). SAA1 and TUBA8 were closely related to the sensitivity of various drugs, which might lead to altered drug sensitivity.

**Conclusion:**

Dysregulation of SAA1, TUBA8 and monocytes are key factors in ankylosing spondylitis with femoral head necrosis.

## Introduction

Ankylosing spondylitis (AS) is a chronic inflammatory disease, primarily involving the mid-axis skeleton. The main characteristic change is sacroiliac arthritis, and the disease occurrence is inextricably linked to genetics (1, 2). AS has a strong genetic predisposition and the lack of efficacy of monoclonal antibodies against different cytokines, highlighting the diversity of type 17 cytokines and immune cells, which is critical to the pathogenesis of spondyloarthritis and AS (3). In AS, the damage to structures due to ankylosing changes and spine tonicity is irreversible (4). Femoral head necrosis, a common clinical disease with a high disability rate, is caused by factors such as excessive alcohol consumption and the heavy use of short-term high-dose glucocorticoids (5). In the disease process of femoral head necrosis, the blood supply is not sufficient to maintain the needs of bone metabolism, and the early treatment of the disease is mainly through conservative treatment, and in case of failure of conservative treatment, surgical treatment is required (6). Bukowski et al. evaluated 174 patients with AS who underwent total hip arthroplasty and demonstrated an incidence of the posterior hip dislocation of approximately 8% over 20 years (7). However, the potential diagnostic biomarkers for AS combined with osteonecrosis of the femur, a disease that has a significant impact on the quality of life of patients, remain largely unknown.

Hypoxia has been reported in several inflammatory diseases. Hypoxia is an important feature in pathological and physiological immune responses, and its effects on inflammation and immunity may vary depending on the specific immune processes and microenvironment. In pathological conditions, chronic inflammation, infection, and tissue ischemia, pathological hypoxia can lead to disease progression and tissue dysfunction through an imbalance in immune cell regulation (8). A study by Kerber et al. noted that hypoxia is a common feature of inflammation, while hypoxia-inducible factor (HIF) is a condition for cellular adaptation verification and hypoxic tensor, and that myeloid HIF-2α and HIF-1α play opposite roles in acute colitis (9). The human response to hypoxia is mediated by HIF, which activates the upregulation of the expression of a set of hypoxia-adapted genes, while HIF-dependent genes contribute to nutrient absorption, immune regulation, and repair of epithelial barrier function, among other roles, under physiological conditions (10). HIF acts as a transcription factor that can play an important role in human cells in a hypoxic environment. HIF-α can directly regulate the expression of immune genes, including macrophages, dendritic cells, neutrophils, B cells, and T cells (11).

Monocytes are a subtype of macrophage progenitors and dendritic cells whose primary role is to assume the role of circulating sensors for environmental changes and responses to disease (12). Recent studies have shown that the synovial fluid of arthritis in psoriatic arthritis is predominantly dominated by monocytes/macrophages, which contain three distinct cell populations, and that classical monocytes/macrophages are reduced in psoriatic arthritis compared to osteoarthritis or rheumatoid arthritis (13). It has also been shown that macrophages dominate synovial tissue and are the main immune cell population in osteoarthritis of the knee, exhibiting expression of synovial fluid-associated β1 (TGFβ1) and elastase or transforming growth factor, and that the expression levels of TGFβ1 and elastase show a trend towards a significant correlation with the severity of osteoarthritis of the knee on imaging (14). The exact mechanism of the role of monocytes/macrophages in AS, especially in AS combined with femoral head necrosis, is not clear to us.

Here, we searched for the role played by hypoxia-related genes in AS by performing label-free protein profiling of six ligamentous tissues from AS with femoral head necrosis and that without AS. Moreover, through a complex and precise bioinformatics approach, we searched for immune cells associated with AS in an attempt to find a new path for immunotherapy for AS. We also used data from routine blood tests to examine the results of our analysis. In addition, the drug sensitivity analysis was performed to provide a new basis of reference for the treatment of AS. The main objective of our study was to analyze the proteomic data of AS combined with femoral head necrosis and to find the genes closely associated with this disease through precise bioinformatics analysis, providing a new reference basis for the early diagnosis and treatment of this disease.

## Materials And Methods

### Sample Source

We collected femoral head ligament tissues for pathological testing from patients who underwent an intraoperative artificial hip replacement at the First Clinical Affiliated Hospital of Guangxi Medical University between 2018 and 2019. This study was approved by the ethical review of the First Clinical Affiliated Hospital of Guangxi Medical University, as per the Declaration of Helsinki of the World Medical Congress. Inclusion criteria for this study were as follows: individuals with AS combined with femoral head necrosis were categorized into the experimental group, and those only with femoral head necrosis were categorized into the control group. Exclusion criteria for this study were: patients with combined rheumatoid arthritis, systemic lupus erythematosus, and tumors. A total of 12 patients were analyzed using a randomized method, with six patients in the experimental group and 6 in the control group. In our study, we used tissues from adults for the experiments and did not use tissues from minors for the experiments. Informed consent was obtained from all participants and/or their legal guardians.

### Label-Free Quantification (LFQ) Protein Park Analysis

In our study, we quantified the proteins using the LFQ method to obtain accurate differences in the proteins of ligaments with AS combined with femoral head necrosis versus those only with femoral head necrosis. First, the samples were lysed, followed by mixing the radio-immune precipitation assay lysate with protease inhibitor and phenylmethylsulfonyl fluoride, which was placed on ice to pre-cool and obtain the working solution. Then the tissue samples were added to 1000 µL of working solution and mixed thoroughly, and sonicated in an ice bath for 5 min until they were fully dissolved. Subsequently, after centrifugation, a 96-well plate was added and shaken at 37°C. The protein concentration of the sample was calculated from the standard protein using the standard curve. Then, we performed acetone precipitation, protein redissolution, reduction, alkylation, enzymatic digestion, sodium deoxycholate removal, peptide desalting, nano-ultra performance liquid chromatography separation, plasmapheresis 120 min/sample, Maxquant analysis and label-free quantification, protein abundance distribution detection, and quantitative replication. All protein expressions of the experimental and control groups were obtained.

### Analysis of Differentially Expressed Proteins

In this study, we used the programming language R (version 4.0.2) to analyze the differentially expressed proteins to find the differentially expressed proteins in the experimental group versus the control group. We identified differentially expressed proteins between the two groups using the “ggplot2” package, the “limma” package, and the “pheatmap” package, with cut-off values. The cut-off value was set to |logFC| > 0.1, adjusted P < 0.05. Subsequently, we constructed a heat map of differentially expressed proteins, plotting the top 50 differentially significant up and down-regulated proteins. Also, we constructed volcano maps of differential proteins.

### Weighted Gene Co-Expression Network Analysis (WGCNA)

WGCNA is an advanced bioinformatics analysis that is widely used in biological lineage information to find the biological modules most associated with the disease (15, 16). A gene co-expression network is an undirected graph, where each node represents a gene and can be established by observing the gene pairs that produce similar expressions between different samples. The principle followed is that two co-expressed genes should vary in the same pattern in different samples. The subsequent construction of scale-free networks, complex networks with a class of features, is typically characterized by the fact that most nodes in the network are connected to only a few nodes. All the detected proteins were analyzed to construct a gene-to-gene similarity network, identify the network modules, cluster the co-expressed genes, explore the association of external modules with gene expression, identify the hub genes in the modules, and define the similarity matrix of gene co-expression using the formula (Sij)^unsigned ^=|cor(i,j)|. The command R automatically selects the most suitable soft threshold β for the optimal slice. Finally, disease-related modules, as well as dynamic shear tree results, were obtained.

### Gene Ontology (GO) and KEGG Pathway Enrichment Analyses

We performed GO and KEGG pathway enrichment analyses for all differentially expressed proteins to gain insight into the function of these differential proteins between the AS and control groups. We used the “colorspace” package, “stringi” package, “ggplot2” package, “digest” package, “GOplot” package, “DOSE” package, “clusterProfiler” package, and “enrichplot” package to analyze the differentially expressed proteins. We set the screening conditions as P < 0.05 and adjusted-P < 0.05. Subsequently, the results of GO and KEGG enrichment analyses were obtained.

### Hypoxia-Associated Genes and Ankylosing Spondylitis Proteins Take Intersections to Construct a Protein-Protein Interaction Network (PPI)

Here, all hypoxia-related datasets in humans were downloaded from the Gene Set Enrichment Analysis database to gain insight into the role of hypoxia-related genes in AS. Subsequently, intersections of the differentially expressed proteins obtained from the screen, the module with the highest disease relevance in WGCNA, and the hypoxia-related genes were taken, and the differentially expressed proteins with the highest disease relevance in AS were obtained. Finally, we imported all the differentially expressed proteins into the STRING (https://www.string-db.org/) database to obtain the protein-protein interaction network, which was finally imported into Cytoscape (version 3.8.0) software for visualization.

### Immune Cell Correlation Analysis

A quantitative analysis of differential expression of immune cells was performed using the CIBERSORT (17, 18) software on the protein expression matrix to analyze the relationship between these proteins and immune cells in AS. Subsequently, two-paired samples t-test was performed both for the experimental and control groups using IBM SPSS Statistics 25, and P < 0.05 was considered statistically significant. We also performed correlation analysis of gene and immune cell content with the gene expression separately for the intersecting two genes.

### Drug Sensitivity Analysis

To analyze the relationship between AS and drug sensitivity, all data related to drug sensitivity was downloaded from the CellMiner database (version: 2021.1, database: 2.6). Subsequently, we used the programming language R (x64, version 4.0.2) to analyze all protein gene expression information and drug sensitivity data using the “impute” (19), “limma” (20), “ggplot2” (21), and “ggpubr” packages (22).

### Blood Routine Data Validation

We performed a statistical analysis of monocytes, neutrophils, eosinophils and erythrocyte sedimentation rates in routine blood examinations of 502 AS and 162 normal controls from the First Clinical Affiliated Hospital of Guangxi Medical University in order to examine the differences in immune cells analyzed by bioinformatics. This study was approved by the Ethics Committee of the First Clinical Affiliated Hospital of Guangxi Medical University and was in accordance with the requirements of the Declaration of Helsinki of the World Medical Assembly.

## Results

### Label-Free Quantification Protein Park Analysis

**Figure 1:**
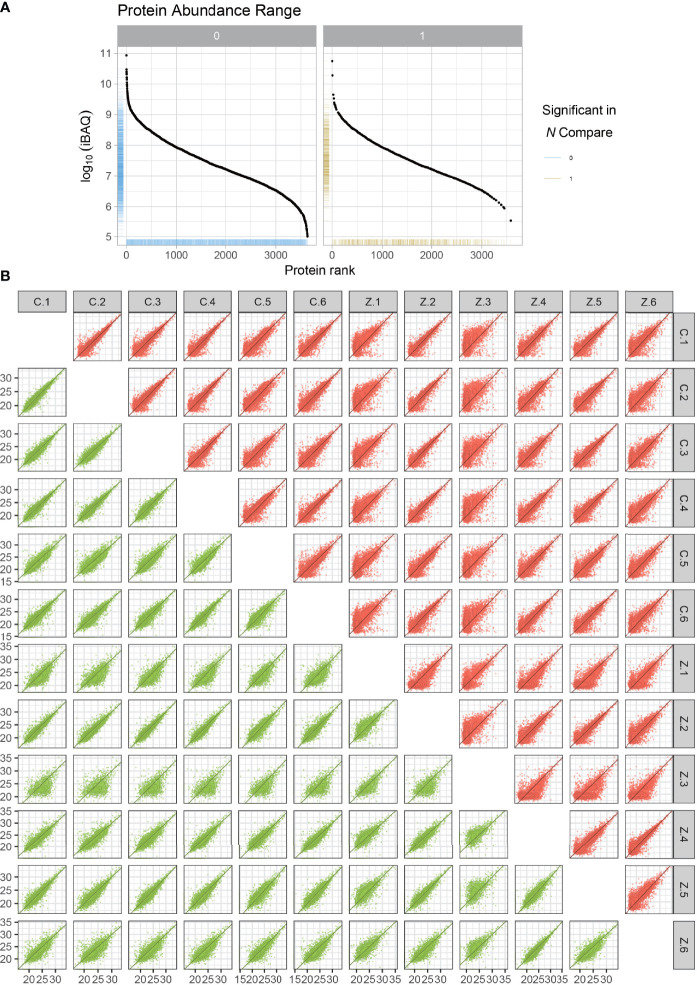
Protein abundance plots and quantitative correlation analysis between samples. The plot of protein abundance is indicated in **(A)** The plot of quantitative correlation analysis between samples is indicated in **(B)** The plot of protein abundance is indicated in **(B)** The plot of protein abundance is indicated in **(B)**.

We randomly selected six ligament specimens from AS combined with femoral head necrosis and six from non-obligatory spondylitis with femoral head necrosis for LFQ protein park analysis. After sample lysis, BCA quantification, acetone precipitation, protein re-solubilization, reduction, alkylation and enzymatic digestion, peptide desalting, we obtained the expression of all proteins. As illustrated in the protein abundance plot (**Figure 1A**) obtained from the protein park analysis, our results using LFQ protein park analysis are plausible. In contrast, we also obtained a correlation heat map of protein expression for all samples (**Figure 1B**). We obtained the expressions of all the proteins expressed for subsequent analysis. From the protein abundance and distribution maps we can find that the quantification and abundance of the proteins we measured meet the requirements of the analysis.

### Analysis of Differentially Expressed Proteins

**Figure 2:**
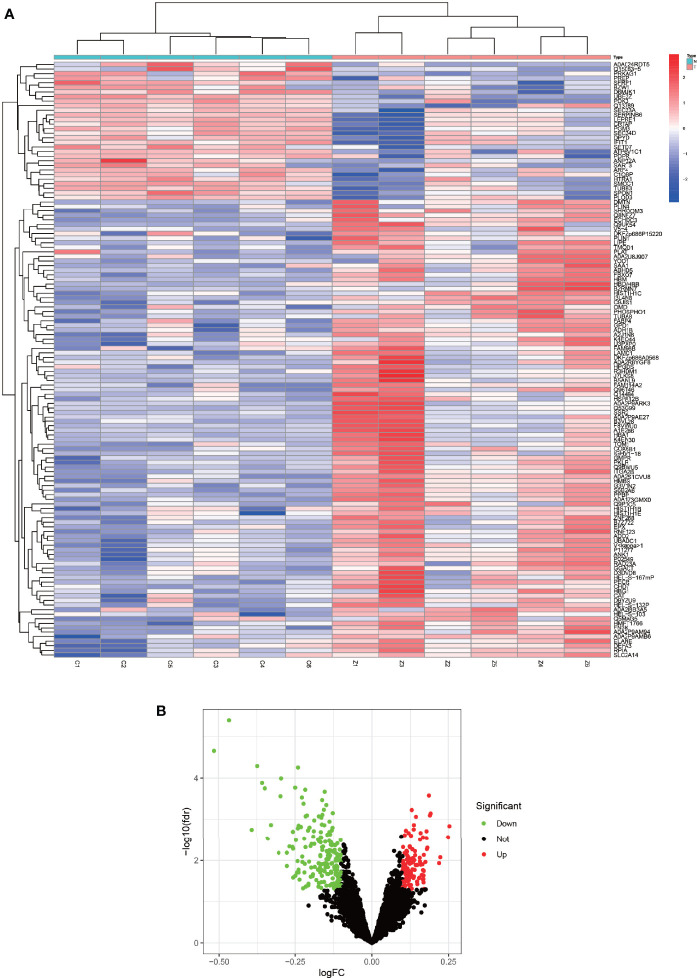
Heat map and volcano map of differentially expressed proteins. **(A)** represents a heat map of the differentially expressed proteins, with the highly expressed proteins in red and the lowly expressed proteins in blue. **(B)** represents a volcano plot of differentially expressed proteins, with high expression proteins in red, low expression proteins in green, and proteins with insignificant differences in black.

R was utilized for differentially expressed protein analysis for a total of 3477 detected proteins. After screening, the top 100 most significantly differentially expressed proteins were obtained. Using the heat map (**Figure 2A**), we can detect that a large portion of proteins was expressed higher in the experimental group than in the control group. Red squares indicate highly expressed proteins and blue squares indicate lowly expressed proteins. Also, we constructed the volcano plot of differentially expressed proteins (**Figure 2B**). Red dots indicate highly expressed proteins and green dots indicate lowly expressed proteins. Details of the differentially expressed proteins can be found in **Table 1**.

**Table 1:** Details of differentially expressed proteins.

id	logFC	AveExpr	t	P.Value	adj.P.Val	B
S6B2A6	-0.46513	4.526695	-7.36086	4.01E-06	0.01394	4.250422
A0A2S1CVU8	-0.51411	4.593434	-6.28385	2.20E-05	0.038297	2.830983
Q9BWU5	-0.37355	4.476655	-5.78499	5.12E-05	0.04836	2.106727
Q8NFZ7	-0.24071	4.388982	-5.73661	5.56E-05	0.04836	2.034214
ECHDC3	-0.29587	4.431591	-5.39184	0.000102	0.070846	1.505964
G3V1N2	-0.35757	4.596005	-5.25257	0.000131	0.073906	1.286953
PPBP	-0.24985	4.372257	-5.10773	0.00017	0.073906	1.055811
A0A173GMX0	-0.34875	4.433052	-5.08563	0.000177	0.073906	1.020257
DMTN	-0.21715	4.370161	-5.04216	0.000191	0.073906	0.95007
I3L4N8	-0.1539	4.393405	-4.98054	0.000214	0.07445	0.850087
UBE2Z	0.187355	4.447355	4.866779	0.000264	0.078792	0.663962
ITGA2B	-0.29768	4.409576	-4.84496	0.000275	0.078792	0.628038
V<kappa>1	-0.22814	4.654721	-4.80751	0.000295	0.078792	0.566212
FN3K	-0.16228	4.602079	-4.72962	0.000341	0.084566	0.436971
ADD2	-0.22264	4.573189	-4.62132	0.000417	0.096654	0.255822
FBXO7	-0.15647	4.516789	-4.44163	0.000585	0.114422	-0.04829
D6MJK1	0.193114	4.424852	4.331283	0.000722	0.115983	-0.2371
HMFT1766	-0.16133	4.376251	-4.29307	0.000777	0.115983	-0.30282
SPON1	0.190578	4.50482	4.275199	0.000804	0.115983	-0.33362
A0A2P9AM94	-0.21126	4.389602	-4.27503	0.000804	0.115983	-0.33391
HMBS	-0.21601	4.572069	-4.25145	0.000841	0.115983	-0.3746
C9JIS1	-0.17299	4.462089	-4.25034	0.000843	0.115983	-0.37652
ANK1	-0.16455	4.86809	-4.21288	0.000906	0.116661	-0.44128
RNF123	-0.18781	4.531046	-4.12952	0.001064	0.132074	-0.58597
PHOSPHO1	-0.16301	4.336236	-4.01126	0.001337	0.141545	-0.79242
LIPE	-0.32857	4.477508	-3.99743	0.001373	0.141545	-0.81665
ABHD5	-0.1552	4.3137	-3.99313	0.001385	0.141545	-0.8242
A0A2I8B3A5	-0.23868	4.448785	-3.97886	0.001424	0.141545	-0.84921
SMOC1	0.253883	4.520326	3.96439	0.001464	0.141545	-0.8746
GGACT	-0.23096	4.458569	-3.94278	0.001527	0.141545	-0.91256
GMPR	-0.20302	4.455492	-3.92581	0.001578	0.141545	-0.94238
PECR	-0.2037	4.351722	-3.91787	0.001603	0.141545	-0.95635
HBM	-0.2411	4.351427	-3.90233	0.001652	0.141545	-0.9837
Q9UK54	-0.39134	4.460414	-3.85814	0.001801	0.141545	-1.06156
ELANE	-0.20821	4.584311	-3.84035	0.001864	0.141545	-1.09295
HIST1H1C	-0.18924	4.398424	-3.82047	0.001938	0.141545	-1.12804
PRKAG1	0.17956	4.351726	3.818012	0.001947	0.141545	-1.13239
A2J1N8	-0.25732	4.471366	-3.80398	0.002001	0.141545	-1.15718
P11277	-0.16653	4.815259	-3.79534	0.002035	0.141545	-1.17246
A0A024RDT5	0.160373	4.373757	3.765115	0.002159	0.144673	-1.22591
UBADC1	-0.19087	4.548922	-3.66209	0.002642	0.156702	-1.40854
HTRA1	0.249471	4.666547	3.643828	0.002739	0.156702	-1.44096
P02549	-0.16708	4.833201	-3.64173	0.00275	0.156702	-1.44469
SETD7	0.167835	4.503148	3.63792	0.002771	0.156702	-1.45146
RPIA	-0.16474	4.622587	-3.6336	0.002794	0.156702	-1.45912
TUBB3	0.17901	4.621359	3.621166	0.002863	0.157823	-1.48122
HBA1	-0.34109	4.435741	-3.60795	0.002939	0.157823	-1.50471
GPD1	-0.22067	4.658329	-3.60591	0.00295	0.157823	-1.50833
ADH1B	-0.17538	4.809187	-3.58026	0.003103	0.160847	-1.55394
PDK3	0.157371	4.402394	3.579208	0.003109	0.160847	-1.55581
TOM1	-0.24781	4.449499	-3.56753	0.003182	0.160847	-1.57658
COX6B1	-0.17192	4.317347	-3.52811	0.003438	0.168366	-1.64673
FAM114A2	-0.152	4.361464	-3.51857	0.003503	0.169173	-1.66371
SHROOM3	-0.16721	4.376497	-3.50229	0.003617	0.170139	-1.69269
PKLR	-0.18776	4.542552	-3.45369	0.00398	0.180261	-1.77924
D9YZU9	-0.19665	4.650619	-3.45222	0.003992	0.180261	-1.78186
DEFA3	-0.19995	4.596871	-3.39177	0.004497	0.180502	-1.88957
FABP4	-0.26874	4.517331	-3.38023	0.0046	0.180502	-1.91013
TUBA8	-0.15917	4.395048	-3.37592	0.00464	0.180502	-1.9178
Q96T46	-0.25865	4.413787	-3.36416	0.004748	0.180502	-1.93876
HEL-S-132P	-0.21653	4.478612	-3.3612	0.004776	0.180502	-1.94403
Q9P1C5	-0.16264	4.352547	-3.35936	0.004794	0.180502	-1.94731
SFRP1	0.181105	4.478018	3.345032	0.004931	0.182392	-1.97284
SAA1	-0.25704	4.454283	-3.31768	0.005204	0.18849	-2.02158
ANP32A	0.179363	4.42283	3.30616	0.005324	0.190747	-2.04209
B7Z722	-0.16188	4.471284	-3.28087	0.005596	0.190747	-2.08714
HBD/HBB	-0.27736	4.40318	-3.20058	0.006556	0.193062	-2.23004
J7LKS8	-0.3049	4.38905	-3.1971	0.006601	0.193062	-2.23623
ATP6V1C1	0.169523	4.406746	3.13737	0.007426	0.201636	-2.34239
LAMC1	-0.16807	4.384278	-3.13016	0.007532	0.201636	-2.35519
HEL-S-167mP	-0.16778	4.334492	-3.0998	0.007996	0.205946	-2.40907
K4EQ44	-0.22706	4.55777	-3.09145	0.008129	0.20782	-2.42388
LEPRE1	0.223088	4.696041	3.072721	0.008434	0.209469	-2.45709
ARF4	0.155964	4.60485	3.055561	0.008724	0.210647	-2.48749
PLIN1	-0.24252	4.712873	-2.96306	0.010464	0.217236	-2.65103
SERPINB6	0.156912	4.68829	2.950401	0.010728	0.218394	-2.67334
A0A2P9AMB6	-0.20484	4.680641	-2.94838	0.01077	0.218394	-2.6769
PGM3	0.174582	4.579072	2.938393	0.010984	0.218394	-2.69451
EPX	-0.22647	4.416321	-2.9369	0.011016	0.218394	-2.69714
Q53G99	-0.15021	4.299872	-2.90759	0.011668	0.223388	-2.74874
CRTAP	0.219215	4.664599	2.904973	0.011728	0.223388	-2.75333
Q14484	-0.17371	4.368887	-2.9016	0.011806	0.223388	-2.75926
U3PXP0	-0.15042	4.527014	-2.90103	0.011819	0.223388	-2.76026
CHD7	-0.15117	4.521314	-2.8952	0.011955	0.223388	-2.77052
B3VL28	-0.21876	4.362942	-2.88561	0.012182	0.223388	-2.78736
HSPA12B	-0.16956	4.34589	-2.88484	0.0122	0.223388	-2.78872
SLC2A14	-0.15735	4.497632	-2.88456	0.012207	0.223388	-2.78921
HBG1	-0.1627	4.434754	-2.87601	0.012413	0.224322	-2.80423
PREP	0.165065	4.72285	2.870906	0.012538	0.224322	-2.81318
PLOD3	0.159294	4.4533	2.86168	0.012766	0.226475	-2.82936
IGHV1-18	-0.27727	4.410019	-2.82394	0.013745	0.234051	-2.89546
OMD	-0.18629	4.466139	-2.79694	0.01449	0.238826	-2.94264
CAT	-0.15091	4.668516	-2.78457	0.014844	0.241184	-2.96424
HIST1H1B	-0.19771	4.617262	-2.77905	0.015005	0.242661	-2.97386
HIST1H1E	-0.16926	4.798429	-2.77522	0.015118	0.24335	-2.98054
Q5MAG5	-0.17594	4.420235	-2.71804	0.016899	0.262889	-3.07995
F8VWU0	-0.23918	4.352574	-2.71664	0.016946	0.262889	-3.08239
Q15063-5	0.166717	4.37575	2.706503	0.017283	0.26357	-3.09996
B2RMN7	-0.16168	4.347062	-2.70203	0.017434	0.26471	-3.10771
SEC24D	0.169451	4.599311	2.683617	0.018069	0.269646	-3.13958
HEL-S-103	-0.1541	4.646185	-2.67057	0.018533	0.271647	-3.16211
RAD23A	-0.16841	4.413893	-2.64289	0.019555	0.271647	-3.20985
B5ANL9	-0.23888	4.34086	-2.63904	0.019702	0.271647	-3.21649
DKFZp686A0568	-0.22009	4.434658	-2.63806	0.019739	0.271647	-3.21818
A0A2U8J907	-0.24425	4.448972	-2.63584	0.019825	0.271647	-3.222
SSR2	-0.19211	4.360938	-2.58286	0.021964	0.282364	-3.31292
C1QBP	0.163145	4.652796	2.569869	0.022521	0.282364	-3.33513
PDPR	0.168495	4.505429	2.55104	0.023354	0.28592	-3.36727
D3DVD8	-0.16314	4.352605	-2.54409	0.023668	0.28787	-3.37911
PLIN4	-0.24959	4.43851	-2.54371	0.023686	0.28787	-3.37976
YOD1	-0.19351	4.448915	-2.53024	0.024308	0.291443	-3.40268
R3HDM1	-0.19942	4.344243	-2.51476	0.025042	0.296061	-3.42898
K4EN30	-0.25688	4.379277	-2.4907	0.026226	0.304314	-3.46975
BZW1	0.155493	4.496649	2.485513	0.026489	0.304314	-3.47853
DPYD	0.154103	4.603036	2.476371	0.026957	0.304314	-3.49398
SART3	0.167974	4.385702	2.446221	0.028558	0.308371	-3.54479
Q13789	0.166736	4.497566	2.431673	0.029362	0.308825	-3.56923
A0A2P9ARK3	-0.2361	4.348022	-2.43082	0.02941	0.308825	-3.57066
A0A2R8YGF8	-0.18128	4.35006	-2.41776	0.030152	0.313892	-3.59256
ZNF268	-0.15598	4.659183	-2.36521	0.033322	0.323631	-3.68023
V5-4	-0.1649	4.284444	-2.35741	0.033819	0.325728	-3.69319
IFIT1	0.168398	4.526224	2.349233	0.034347	0.328783	-3.70674
TMOD1	-0.17271	4.411488	-2.32775	0.035772	0.33122	-3.74228
A1E286	-0.2067	4.338946	-2.30847	0.037098	0.337378	-3.77407
FAM96B	-0.20726	4.612319	-2.26416	0.040326	0.344964	-3.84672
DKFZp686P15220	-0.21578	4.527072	-2.22475	0.043417	0.350078	-3.91085
PLAT	-0.17416	4.465945	-2.22423	0.043459	0.350078	-3.9117
A0A2P9AE27	-0.16522	4.357455	-2.18887	0.046422	0.364065	-3.9688
HPGDS	-0.22456	4.451665	-2.17342	0.047776	0.371302	-3.99364
SEC23A	0.170258	4.700001	2.152839	0.049636	0.375373	-4.02659

### WGCNA

**Figure 3:**
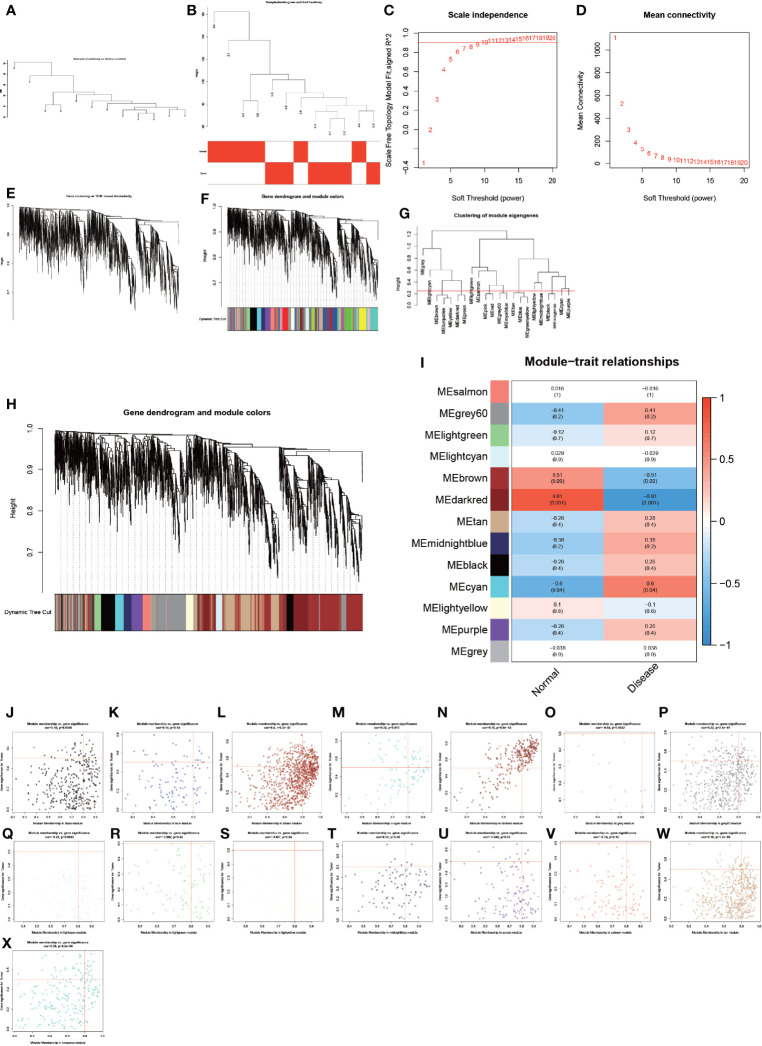
Results of weighted gene co-expression network analysis. **(A–X)** show the entire WGCNA process, from sample clustering to correlation analysis, looking for the genes in the modules most associated with the disease.

To analyze these detected proteins from different analytical perspectives, we used the complex and precise method of WGCNA for analysis (**Figure 3**). The WGCNA method is an advanced bioinformatics method for screening biomarkers of diseases with high accuracy (23, 24). We used the WGCNA method to screen the genes of the modules with the highest correlation to the disease in order to obtain more accurate biomarkers for AS combined with femoral head necrosis. We first performed a cluster analysis on all specimens and subsequently let R choose the most appropriate soft threshold β. Here, our soft threshold β was set to 8. Then, dynamic shear trees, as well as constructed modules, were constructed with the highest correlation with disease and control groups (**Figures 3A–I**). We also analyzed the relationship of gene expression values of module-associated genes, visualizing them as dot plots (**Figures 3J–X**).

### GO and KEGG Pathway Enrichment Analysis

**Figure 4:**
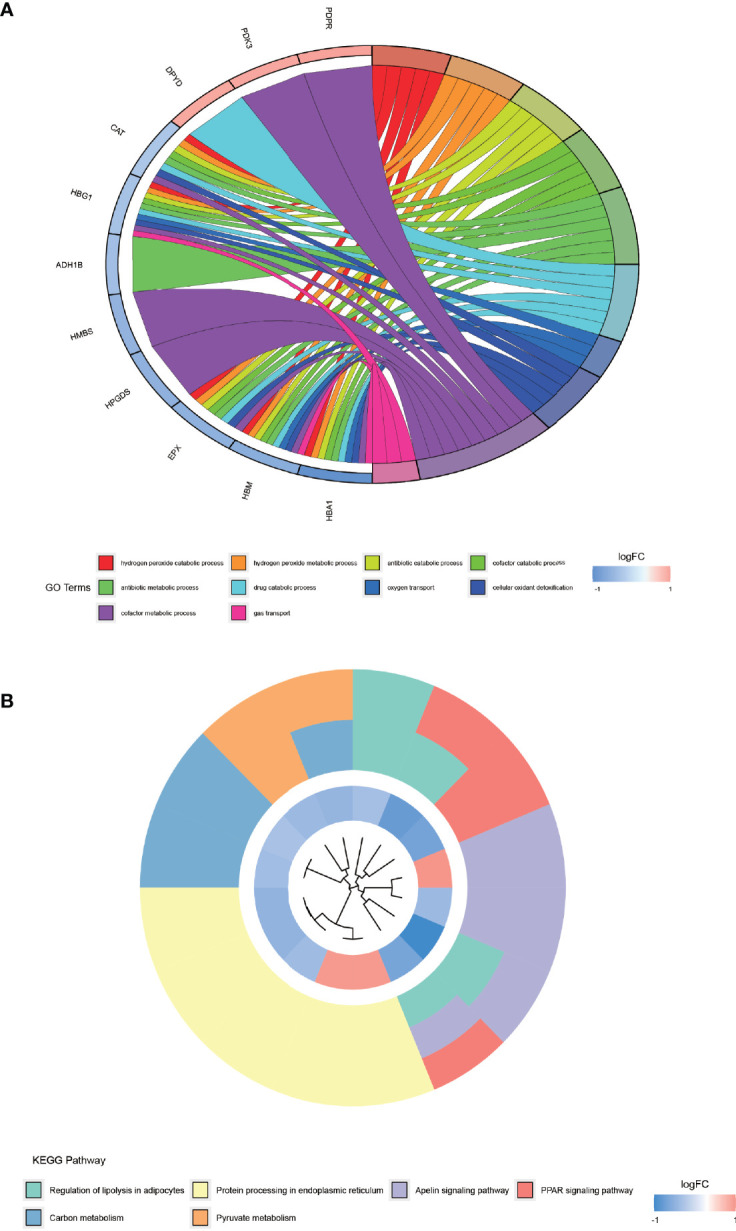
GO enrichment analysis and KEGG pathway enrichment analysis of differentially expressed proteins. **(A)** shows the top 10 entries of GO enrichment analysis for differentially expressed proteins. **(B)** shows the KEGG pathway enriched by the differentially expressed proteins.

The results of GO enrichment analysis (**Figure 4A**) demonstrated that the top 10 GO terms are mainly distributed in hydrogen peroxide catabolic process, hydrogen peroxide metabolic process, antibiotic catabolic process, cofactor catabolic process, antibiotic metabolic process, drug catabolic process, oxygen transport, cellular oxidant detoxification, cofactor metabolic process, and gas transport. KEGG pathway (**Figure 4B**) is primarily distributed in the regulation of lipolysis in adipocytes, protein processing in the endoplasmic reticulum, apelin signaling pathway, peroxisome proliferator-activated receptor (PPAR) signaling pathway, carbon metabolism, and pyruvate metabolism. These differentially expressed proteins are enriched indistributed in the regulation of lipolysis in adipocytes pathway, which provides a new reference for the lower than normal weight of AS patients.

### Hypoxia-Associated Genes and Ankylosing Spondylitis Proteins Take Intersection, Construction of PPI

**Figure 5:**
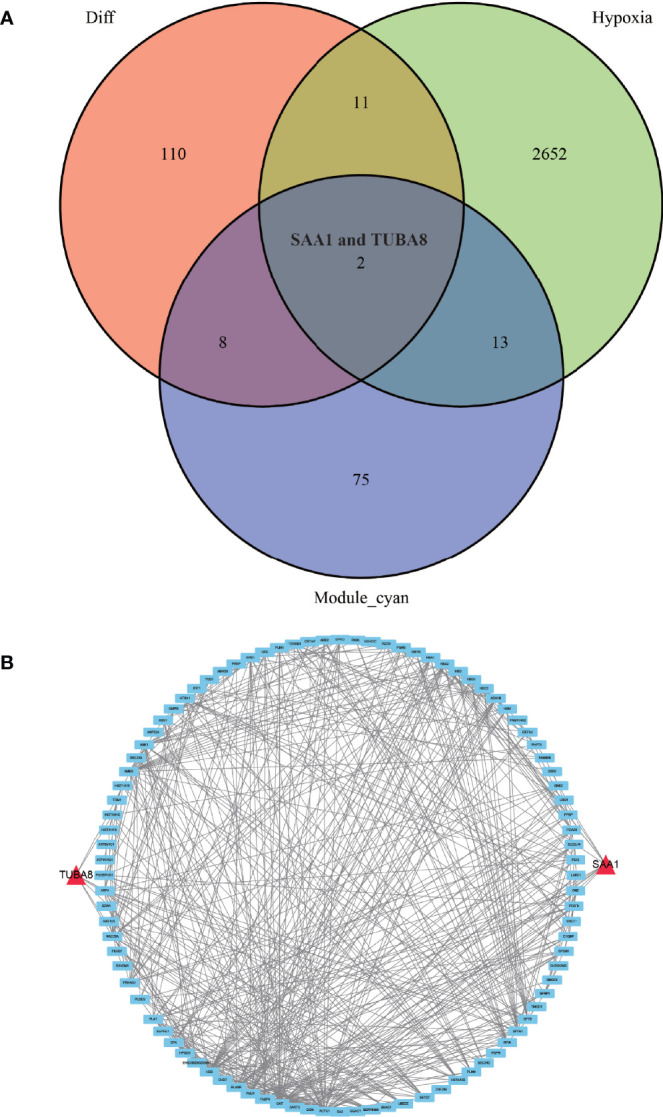
Wayne diagram and protein-protein interaction network diagram. **(A)** shows the results of taking the intersection of the differentially expressed protein, the hypoxia-associated gene, and the gene from the module most associated with obligatory spondylitis. **(B)** shows a protein-protein interaction network in which TUBA8 and SAA1 are closely associated with multiple significantly different proteins.

We conducted an in-depth study using a dataset of hypoxic genes in order to analyze the specific role of hypoxia in AS. We de-intersected the differentially expressed proteins, hypoxia-associated genes, and modules with the highest relevance to ankylosing inflammation, and finally, obtained only two proteins, TUBA8 and SAA1, that fulfilled the three conditions (**Figure 5A**). We found from the constructed protein-protein interaction network (**Figure 5B**) that TUBA8 and SAA1 are each closely associated with several genes, which may influence the expression of these genes and jointly participate in regulating the disease process of AS combined with femoral head necrosis.

### Immune Cell Correlation Analysis

**Figure 6:**
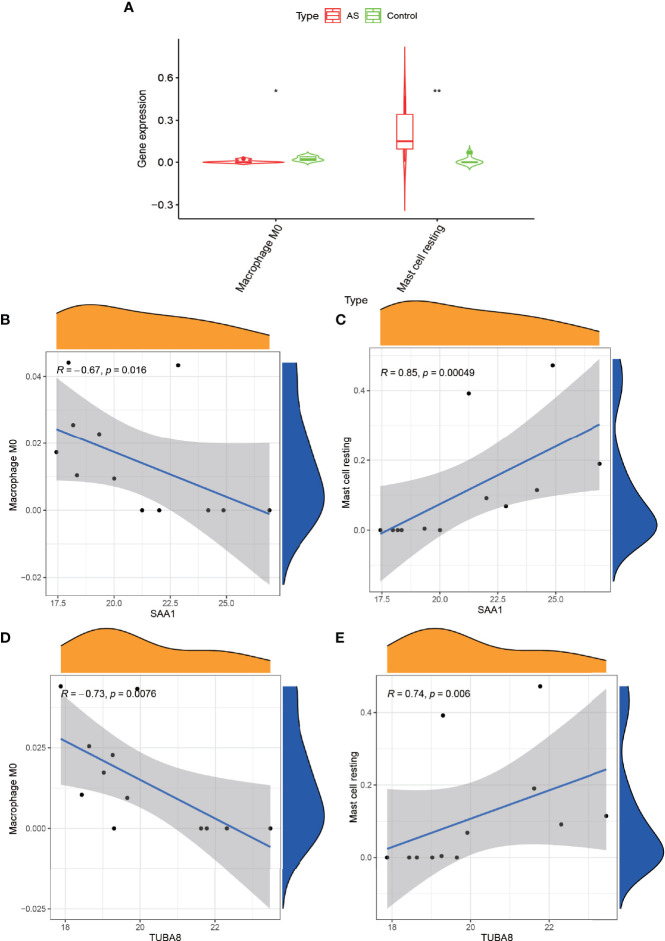
Immune cell correlation analysis graph. **(A)** shows the immune cells obtained after analysis by CIBERSORT software at significant differences, “*” indicates P < 0.05, “**” indicates P < 0.01. **(B, C)** represent the correlation analysis of gene expression of SAA1 with Macrophage M0 and Mast cell resting. Figure **(D, E)** represent the correlation analysis of gene expression of TUBA8 with Macrophage M0 and Mast cell resting.

We obtained the immune cell content of all samples after a comparative analysis of all protein expressions using CIBERSORT software. After the t-test of two paired samples in IBM SPSS Statistics 25, we found that macrophage M0 and resting mast cells were quite significantly different in the experimental and control groups (**Figure 6A**) (P < 0.05). Also, we analyzed the correlation between these two genes and the content of immune cells and found that SAA1 showed a significant negative correlation with macrophage M0 (**Figure 6B**) (R = –0.67, P = 0.016) and a strong positive correlation with resting mast cells (**Figure 6C**) (R = 0.85, P = 0.00049). TUBA8 showed a significant negative correlation with macrophage M0 (R = –0.73, P = 0.0076, **Figure 6D**), and a significant positive correlation with resting mast cells (R = 0.74, P = 0.006, **Figure 6E**). And the dysregulation of these immune cells may be one of the factors that promote the progression of AS combined with femoral head necrosis.

### Drug Sensitivity Analysis

**Figure 7:**
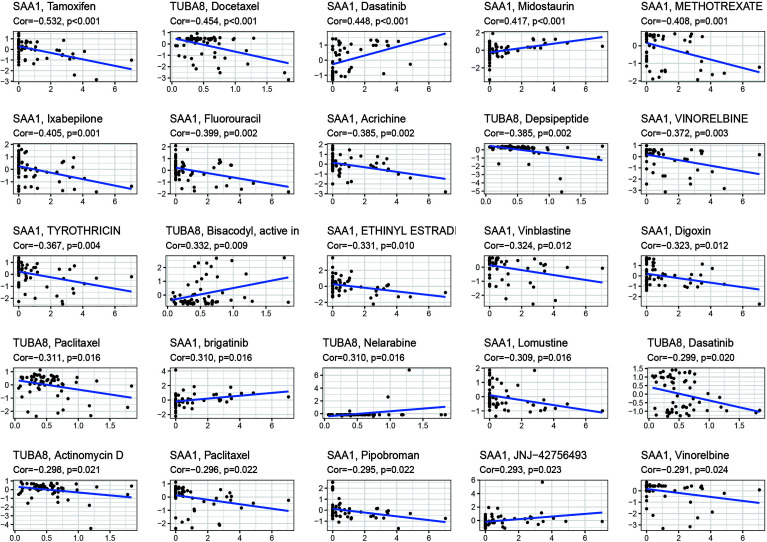
Drug sensitivity analysis. The figure shows that SAA1 and TUBA8 showed different sensitivity correlations with a number of different drugs, respectively. If the correlation is positive, the higher the expression of this gene, the greater the sensitivity to the drug; if the correlation is negative, the lower the expression of this gene, the greater the sensitivity to the drug.

These drugs were selected for analysis in relation to genes associated with AS combined with femoral head necrosis because we found that many of the drugs used to treat AS were antitumor agents that were later found to improve the condition of AS. Inspired by this, we analyzed the relationship between these two genes, which are extremely closely related to AS combined with femoral head necrosis, and the drug sensitivity of antineoplastic drugs. After the drug sensitivity analysis, we visualized the results (**Figure 7**). We can see from the graph that the SAA1 gene showed a positive or negative correlation with the sensitivity of various drugs, including tamoxifen, dasatinib, midostaurin, and methotrexate. Also, TUBA8 was correlated with the sensitivity of various drugs, including docetaxel, depsipeptide, and paclitaxel. A positive correlation indicates that the sensitivity of the corresponding drug is enhanced with the increase in the gene expression value. A negative correlation indicates that with the increase in the gene expression value, the sensitivity of the corresponding drug is reduced. This has given us novel insights into the use of medications to treat chronic compulsive spondylitis.

### Blood Routine Data Validation

**Figure 8:**
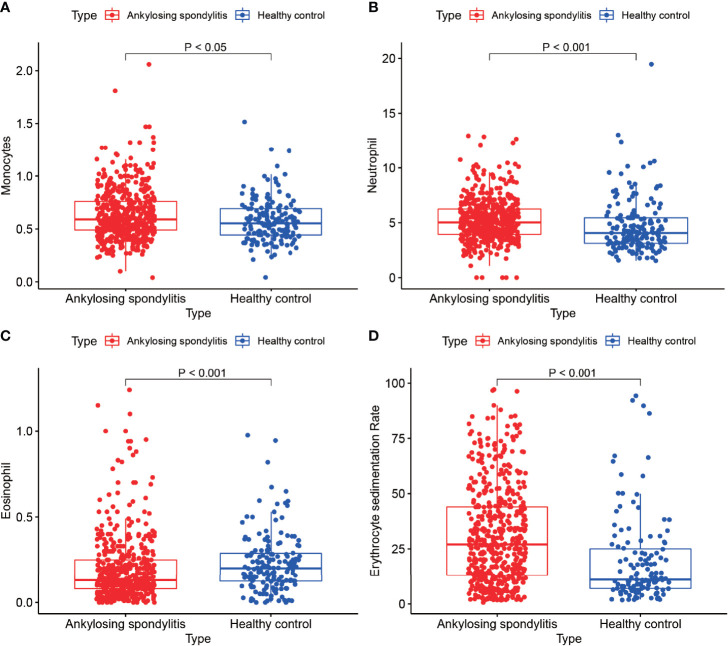
Routine blood tests. **(A–D)** show the results of routine blood tests for macrophages, neutrophils, eosinophils and sedimentation in 502 cases of AS and 162 healthy controls.

We analyzed the monocyte, neutrophil, eosinophil and erythrocyte sedimentation rates in the blood of 502 AS cases and 162 normal controls (**Figures 8A–D**) and found that the monocyte count, neutrophil count and erythrocyte sedimentation rate in the blood of AS cases were higher than those of normal controls, with statistically significant differences (P < 0.05). In addition, we found higher eosinophils in the normal control group than in the AS group, with a statistically significant difference (P < 0.05). This result is consistent with the results of our analysis. Our previous differential analysis of immune cells found significant differences in macrophage M0 between the AS and normal groups, and this result was tested to be accurate by comparing routine blood data from AS patients with normal controls.

## Discussion

Most of the previous studies on AS combined with femoral head necrosis are in the areas of pharmacological treatment of femoral head necrosis and surgical assessment of the efficacy of femoral head necrosis, and little is known about the development of biomarkers for AS combined with femoral head necrosis. Our study quantified proteins from AS combined with femoral head necrosis versus controls in an attempt to find biomarkers for this disease and to provide a new reference for early diagnosis and treatment.

In this study, by analyzing the differentially expressed proteins in hip ligaments with AS with femoral head necrosis and those with femoral head necrosis without AS, GO enrichment analysis revealed that GO entries were mainly enriched in cofactor catabolic process, antibiotic metabolic process, drug catabolic process, oxygen transport, cellular oxidant detoxification, and cofactor metabolic process. Azithromycin, one of the antibiotics, can regulate the disruption of cellular function and intracellular lipid transport, regulation of surface receptor expression, disruption of macrophage phenotype, and autophagy (25). In contrast, the results of KEGG pathway analysis showed that its pathways are mainly distributed in the regulation of lipolysis in adipocytes, protein processing in the endoplasmic reticulum, apelin signaling pathway, PPAR signaling pathway, carbon metabolism, and pyruvate metabolism. It has been shown that matrix metalloproteinase-8 inhibitors have therapeutic potential in the treatment of neuroinflammatory diseases associated with astrocyte reactivity, which may further be associated with the PPAR signaling pathway (26). This is consistent with the results of our study. GO enrichment analysis in this study showed that differentially expressed proteins are also involved in the regulation of immune cells.

Serum amyloid A1 (SAA1) is a protein-coding gene, and the diseases most closely associated with this gene are amyloidosis Aa. Moreover, the results of the study by Lee et al. showed that in a non-pathogenic environment, cytokines from lymphoid cells produce interleukin (IL)-17. The immune cells such as T helper 17 (Th17) cells receive cytokines secreted by neighboring intestinal epithelial cells. SAAs are also capable of leading to a differentiation program of pathogenic pro-inflammatory Th17 cells and act directly with T cells in conjunction with signal transducer and activator of transcription 3-activating cytokines (27). SAA1 expression has been demonstrated as an important link between the mucosal microbial community, T cells, and the tissue environment in patients with inflammatory bowel disease (28). The expression level of SAA1 in mice was inversely correlated with the endotoxin concentration in serum and lung tissues as SAA1 could bind directly to lipopolysaccharide to form a complex; thereby, promoting lipopolysaccharide uptake by macrophages and disrupting the SAA1-lipopolysaccharide interaction with SAA1-derived peptides, possibly exacerbating the degree of inflammation (29). Previous studies have shown that the SAA gene family has been identified as an important biomarker for rheumatoid arthritis, with SAA1 being associated with the acute phase of inflammation (30). This is consistent with the results of our study. In this study, by analyzing the immune cells of the proteins of the hip ligament in AS, we found that it shows a significant correlation with macrophage M0 and resting mast cells. Moreover, SAA1 and monocytes/macrophages showed a significant dysregulated state in AS, which may further contribute to the progression of AS with femoral head necrosis injury. We examined the difference in monocytes/macrophages in AS using routine blood data from 502 AS and 162 normal controls and showed that monocytes/macrophages were significantly higher in the AS group than in the healthy controls. This further tests the results of our analysis. In contrast, our study of drug sensitivity showed a close relationship between SAA1 and that of various drugs such as tamoxifen, dasatinib, midostaurin, and methotrexate. Moreover, the high and low expression of its gene could affect the sensitivity of these drugs quite significantly. This provides a new reference to guide the pharmacological treatment of AS.

Tubulin Alpha 8 (TUBA8) is a protein-coding gene, and diseases closely related to this gene include polymicrogyria with optic nerve hypoplasia. TUBA8 is relatively high in mouse liver. TUBA8 is more strongly expressed in transformed cells compared to non-tumor tissues (31). In contrast, research on TUBA8 has mainly focused on the brain (32–35). In our study, TUBA8 was differentially expressed in AS with femoral head necrosis, and this gene was associated with hypoxia. Moreover, this gene showed a significant correlation with macrophage M0 and resting mast cells, providing new reference information for the later treatment of AS. This is consistent with our study, which showed that TUBA8 and macrophages are significantly dysregulated in AS, and their possible contribution to the progression of AS with femoral head necrosis. Results derived from examination of routine blood data from 502 AS and 162 healthy controls also showed significant differences in macrophages in AS and healthy controls. Moreover, our study showed the correlation of TUBA8 with the sensitivity of several drugs, including docetaxel, depsipeptide, and paclitaxel, and the high and low expression of this gene can significantly affect the drug sensitivity in humans, which provides a new reference for the drug treatment of AS.

Here, we examined the protein expression of hip ligaments with AS with femoral head necrosis as the experimental group and those with femoral head necrosis without AS as the control group using the label-free quantification protein park analysis laboratory for protein detection. The differentially expressed proteins of hypoxia were analyzed by a complex and precise bioinformatics approach to the differentially expressed proteins, the WGCNA approach to the modules most closely related to AS, and the operation of taking intersections of hypoxia-related genes. Furthermore, by analyzing the relationship between these proteins and immune cells, as well as the differential analysis of immune cells we found that monocytes/macrophages differed significantly in the AS and control groups. We examined the differentially expressed genes using two GEO datasets and the test results supported our analysis. We tested the immune cell differential analysis using routine blood data from 502 AS and 162 healthy controls, and the test results supported our analysis. Finally, we also found a strong association between TUBA2 and SAA1 and drug sensitivity of multiple drugs, which provides a basis for new insights into the use of drugs for the treatment of AS.

This study had certain limitations as other studies. First, the inadequacy of the sample size. Considering the analysis of large samples, we only used six pairs of a total of 12 samples for the protein park test, which was insufficient. There were limitations in using routine blood data from 502 AS samples and 162 healthy control samples to examine differential immune cell analysis. Secondly, our study used a large number of laboratory manipulations and experiments for the detection of proteins in the early stage; thus, leaving a gap with the multifaceted and multilevel verification of our experimental results.

## Conclusion

Dysregulation of SAA1, TUBA8 and monocytes are key factors in ankylosing spondylitis with femoral head necrosis.

## Data Availability Statement

The original contributions presented in the study are included in the article/**Supplementary Material**. Further inquiries can be directed to the corresponding author.

## Ethics Statement

The studies involving human participants were reviewed and approved by Ethics Department of the First Clinical Affiliated Hospital of Guangxi Medical University. Written informed consent for participation was not required for this study in accordance with the national legislation and the institutional requirements.

## Author Contributions

JJ, CL, and XZ designed the study. TL, LC, SH, and XS analyze the data. WJ, JC, TC, HL, and YY digital visualization. SW and JZ collected data on routine blood data. JJ wrote and revised the manuscript. CL and XZ revised the manuscript. All authors read and approved the final manuscript. All co-authors participated in the laboratory operation. All authors read and approved the final manuscript.

## Funding

This study was supported by the Youth Science Foundation of Guangxi Medical University, Grant/Award Numbers: GXMUYFY201712; Guangxi Young and Middle-aged Teacher’s Basic Ability Promoting Project, Grant/Award Number: 2019KY0119.

## Conflict of Interest

The authors declare that the research was conducted in the absence of any commercial or financial relationships that could be construed as a potential conflict of interest.

## Publisher’s Note

All claims expressed in this article are solely those of the authors and do not necessarily represent those of their affiliated organizations, or those of the publisher, the editors and the reviewers. Any product that may be evaluated in this article, or claim that may be made by its manufacturer, is not guaranteed or endorsed by the publisher.
