# Supplementary material for: Dysregulation of SAA1, TUBA8 and Monocytes Are Key Factors in Ankylosing Spondylitis With Femoral Head Necrosis
Source: Front Immunol. 2022 Jan 18;12:814278. doi: 10.3389/fimmu.2021.814278 (PMC8812255; doi:10.3389/fimmu.2021.814278)
Supplement: Supplementary file 1 [file DataSheet_1.docx]

The original data link is as follows: https://www.jianguoyun.com/p/DbZPTJcQy93NCRjdupoE.
